# Analysis and computer program for rupture-risk prediction of abdominal aortic aneurysms

**DOI:** 10.1186/1475-925X-5-19

**Published:** 2006-03-10

**Authors:** Clement Kleinstreuer, Zhonghua Li

**Affiliations:** 1 Department of Mechanical and Aerospace Engineering and Department of Biomedical Engineering, North Carolina State University, Raleigh, North Carolina, USA; 2 Endovascular Division, Cordis Corporation (a Johnson & Johnson Company), Miami Lakes, Florida, USA

## Abstract

**Background:**

Ruptured abdominal aortic aneurysms (AAAs) are the 13^th ^leading cause of death in the United States. While AAA rupture may occur without significant warning, its risk assessment is generally based on critical values of the maximum AAA diameter (>5 cm) and AAA-growth rate (>0.5 cm/year). These criteria may be insufficient for reliable AAA-rupture risk assessment especially when predicting possible rupture of smaller AAAs.

**Methods:**

Based on clinical evidence, eight biomechanical factors with associated weighting coefficients were determined and summed up in terms of a dimensionless, time-dependent severity parameter, SP(t). The most important factor is the maximum wall stress for which a semi-empirical correlation has been developed.

**Results:**

The patient-specific SP(t) indicates the risk level of AAA rupture and provides a threshold value when surgical intervention becomes necessary. The severity parameter was validated with four clinical cases and its application is demonstrated for two AAA cases.

**Conclusion:**

As part of computational AAA-risk assessment and medical management, a patient-specific severity parameter 0 < SP(t) < 1.0 has been developed. The time-dependent, normalized SP(t) depends on eight biomechanical factors, to be obtained via a patient's pressure and AAA-geometry measurements. The resulting program is an easy-to-use tool which allows medical practitioners to make scientific diagnoses, which may save lives and should lead to an improved quality of life.

## Background

Abdominal aortic aneurysms (AAAs) are local, irreversible enlargements, affecting 0.4% of people over the age of 50. 15,000 patients die each year from AAA rupture in the US alone. Rupture may occur spontaneously once the induced mechanical wall stress exceeds the local minimum strength of the AAA wall. However, accurate wall stress/strength measurements are not routinely available *in vivo*. Hence, key biomechanical factors (BFs) influencing AAA rupture are employed to determine when elective repair, i.e., open surgery or endovascular repair, is necessary. Several possible AAA-rupture indicators have been proposed, including maximum AAA diameter, expansion rate, mechanical stress, diastolic pressure, asymmetry index, wall stiffness, intraluminal thrombus ratio, saccular index, wall curvature, gender, serum elastin peptide, the ratio of AAA diameter to the third lumbar vertebral, and others. Clearly, the maximum AAA diameter and its expansion rate are the two AAA-rupture risk criteria most frequently employed.

### Maximum diameter

Clinical data shows that the rupture risk is exponentially related to the maximum AAA diameter. Myers et al. [1] stated that for abdominal aortas of 1.9 cm≤*d*_*AA *_≤2.6 cm, the rupture chance for AAAs with maximum diameters less than 4.0 cm, 4.0–4.9 cm, 5.0–5.9 cm, 6.0–6.9 and > 7.0 is 2%, 3.2%, 25%, 35% and 75%, respectively. As indicated, the maximum transverse diameter, *d*_*AAA*,*max *_, is taken as the main criterion for judging the necessity of surgical intervention in asymptomatic AAAs because it is easy to measure. However, there is no consensus for a threshold value regarding the appropriate diameter for intervention, although some investigators regard 5.5 cm as the critical value for elective repair. While it is obvious that a large AAA is more prone to rupture than small ones, there is clinical evidence that small aneurysms may rupture as well. For example, Limet et al. [2] declared that about 12% of AAAs with diameters of 4 to 5 cm ruptured in their clinical investigations. Furthermore, Fillinger et al. [3,4] reported that 10–24% of the ruptured AAAs were 5 cm or less in maximum diameter. Clinically, the maximum AAA transversal diameters of 5 cm for women and 6 cm for men are most commonly used to recommend surgical intervention [5]. Because of the well-known difference in aorta diameter (1.5 cm–2.5 cm) for different patients, the simple criterion of AAA maximum diameter is not accurate to evaluate all patients. As a case in point, the individual-independent parameter, i.e., the ratio  seems better than the maximum AAA diameter alone. Clinical study of Cappeller et al. [6] confirm that threshold values of *χ *= 2.2 and 3.3 for elective repair and possible rupture prediction, respectively, may provide more reasonable recommendations than AAA size alone.

### Expansion rate

Besides the maximum transverse diameter, the expansion rate is another important indicator for AAA rupture. Clinically, a high expansion rate, say from 0.5 cm per year and up, is often associated with a high risk of rupture [7,8]. According to Wolf et al. [9], if the expansion rate reaches more than 0.5 cm/year, elective repair should be considered even if *d*_*AAA*,*max *_< 5 cm. More specifically, Hallin et al. [10] reported that AAA expansion averaged 0.2–0.4 cm/year for AAAs smaller than 4 cm, 0.2–0.5 cm/year for AAAs of 4–5 cm and 0.3–0.7 cm/year for those larger than 5 cm, and the associated rupture risk at four years was 2%, 10% and 22%, respectively. In addition, Brown et al. [11] described that the mean expansion rate in patients with ruptured AAAs was 0.84 cm/year compared to 0.39 cm/year in non-ruptured AAAs. Because expansion rate is a critical key factor related AAA rupture risk, in the following risk assessment program, the weighted factor of expansion rate is relatively large.

### Mechanical stress

The general consensus is that the peak wall stress is the best indicator of AAA rupture, although the maximum AAA diameter and growth rate as well as aneurysm asymmetry are very important and much easier to measure [12]. However, how to define the critical threshold-value and yield stress in different patients is not clear. Because direct stress measurements in AAA patients are not available, *s*oftware packages for structural analysis, such as ANSYS (ANSYS Inc.), ABAQUS (ABAQUS, Inc), ADINA (ADINA R & D, Inc), and FIDAP (Fluent, Inc.) are efficient tools. For example, Di Martino et al. [13] employed the finite-element software FIDAP to simulate fluid-structure interaction in realistic AAA models, and indicated that the complicated AAA geometry would affect the stress distribution considerably. Raghavan et al. [14] used ANSYS to simulate three-dimensionally reconstructed AAA models and found that the 5-cm AAA-diameter criterion as a rupture predictor was not sufficient. To test the validation of wall stress as the key rupture criterion, Fillinger et al. [3,4] did *in vivo *analyses of mechanical wall stress and AAA-rupture risk, and suggested that the peak wall stress seems to be superior to maximum AAA diameter in predicting rupture risk. Thubrikar et al. [15] found that different regions of an AAA had different yield stresses, yield strains and other mechanical properties. Similarly, Raghavan et al. [16] reported that the failure tension of AAA-specimen strips varied regionally with failure stress, i.e., from 33.6 to 235.1 N/cm2. Vorp et al. [17] asserted that a biomechanics-based approach to predict AAA rupture on a patient-specific basis might ultimately prove to be superior to the currently used maximum diameter criterion. Venkatasubramaniam et al. [18] performed a comparative study of aortic wall stress using finite element analysis for ruptured and non-rupture abdominal aortic aneurysms. They indicated that the peak wall stress was significantly higher in the ruptured AAA than in the non-ruptured AAA. In conclusion, accurate estimations of stress and tensile strength in AAA walls are important for predicting aneurysm rupture.

### Diastolic pressure

Hypertension is considered to be a key factor contributing to AAA rupture. It is well known that the maximum blood pressure, i.e., systolic pressure, is the main force to cause AAA-wall deformation. The relationship between the maximum wall stress and systolic pressure is approximately exponential. Nevertheless, an interesting finding is that no significant clinical difference in systolic pressure between non-ruptured and ruptured AAAs exists [19,20]. Thus, although the systolic pressure is the main force to cause AAA-wall stress, it alone cannot be regarded as a predictor for AAA rupture. In contrast, the diastolic pressure is found to be closely associated with AAA rupture. For example, Hatakeyama et al. [19] reported 72% of ruptured AAAs in patients with diastolic hypertension. Cronenwett et al. [20] also asserted that diastolic hypertension must be evaluated to assess the accuracy in predicting small AAA rupture. They defined the diastolic pressure value of 75 mmHg, 90 mmHg and 105 mmHg as the low, middle and high-risk levels for AAA rupture. Powell et al. [21] proposed that the ankle/branchial pressure index (ABPI) is an important prognostic indicator for AAA rupture, and patients with an ABPI below 0.87 have the highest mortality risk. Schewe et al. [22] declared that the AAA expansion rate is significantly correlated with the diastolic pressure and a high diastolic pressure is an important risk factor for both AAA expansion and rupture. Wilson et al. [23] studied the effect of diastolic pressure on AAA rupture and found that a high diastolic pressure may reduce the time to rupture considerably. Presently, the correlation between diastolic hypertension and AAA rupture is accepted by most researchers.

### Asymmetry index

As a result of the local support provided by lumbar vertebrates, most AAAs are asymmetric. Generally, the anterior size is greater than the posterior size with a larger wall thickness at the posterior side than at the anterior side. According to Vorp et al. [24], the shape factor has a substantial influence on the distribution of wall stress within the aneurysm, where the magnitude of the peak stress in the wall increased non-linearly with increasing asymmetry. Hua et al. [25] stated that a simple symmetric model is unreliable in predicting the location and magnitude of peak stresses in most AAAs. Sacks et al. [26] indicated that both AAA surface geometry and hence stress distribution are highly complex and cannot be simulated via simple axisymmetric models. Thus, assessing rupture risk for typical AAAs may require detailed three-dimensional modelling. Finol et al. [5] studied the effect of asymmetry in AAAs under physiologically realistic flow conditions. They graphed the peak wall shear stress and peak wall pressure as a function of aneurismal asymmetry. The effect of asymmetry increases the maximum wall shear stress at peak flow and induces the appearance of secondary flows during the late diastole. Furthermore, mechanical stress concentrations may be triggered by asymmetric and complicated AAA geometries [3,4]. Fillinger et al. [27] also indicated that when matched for age, gender and diameter, ruptured AAAs tend to be less tortuous, yet have greater cross-sectional diameter asymmetry. Both wall thickness and geometry asymmetry affect the stress exhibited by a virtual AAA. Based on fluid-structure interaction analysis, Scotti et al. [28] reported that an asymmetric AAA with regional variations in wall thickness would be exposed to higher mechanical stresses and an increased risk of rupture than a more fusiform AAA with uniform wall thickness. Thus, since most AAAs are asymmetric, a shape index should be taken into account when evaluating AAA rupture.

### Effect of intra-luminal thrombus

An intra-luminal thrombus (ILT) is an accumulation of fibrin, blood cells, platelets, blood proteins and cellular debris adhering to the AAA inner wall. Clinically, 75% of AAAs include thrombi [29]. At present, the effect of ILT on AAA rupture is still controversial [30]. Some investigators think ILT may reduce the stress in the AAA wall. For example, Wang et al. [29] reported that the peak wall stress may be reduced from 6~38% if the ILT-AAA volume ratio ranges from 0.29–0.72. Vorp et al. [31] reported that an ILT could improve the compliance of the wall like a cushion, reduce mechanical stress, and hence was beneficial for preventing AAA rupture. Mower et al. [32] simulated the ILT in an AAA and found that an ILT significantly reduced AAA wall-stress if the ILT became solid. In contrast, some researchers declared that ILTs could accelerate AAA rupture. For example, Wolf et al. [9] found that an increased AAA-ILT volume is associated with a higher likelihood of rapid expansion. They stated that the larger the ILT volume in the AAA cavity, the higher is the possibility of rupture. Cappeller et al. [6] indicated that if the ILT/AAA volume ratio is more than 0.45, the rupture rate becomes very significant. They took the ILT/AAA volume ratio of 0.62 as one possible indicator for AAA rupture. Stenbaek et al. [33] showed that patients with AAAs more than 4 cm and whose ILT area increased by greater than 1.5 cm^2^/year were prone to rupture. They suggested that the growth of ILT may be a better predictor of rupture than AAA diameter. Interestingly, some researchers stated that there is no close relationship between ILT presence and AAA rupture. To prove the point, Schurink et al. [34] tested the blood pressure close to the inner wall of an AAA and found that the pressure is almost the same as that in the lumen. Another question is if ILT volume in the AAA sac is associated with AAA size. According to Pillari et al. [35], for AAAs with *d*_*AAA*,,*max *_>7 cm no change in ILT volume was found with increasing sac diameter; however for the range of 5<*d*_*AAA*,,*max *_<7 cm, the increase in sac diameter was associated with an increase in ILT volume. The impact of the maximum ILT volume before AAA rupture remains controversial. Vorp et al. [36] stated that an ILT can decrease the ultimate AAA strength dramatically. They found that the strength of an AAA with 4 mm ILT decreases 20% compared to an AAA with ILT less than 1 mm. Hypoxia, i.e., oxygen deficiency caused by an ILT, is the main reason for wall strength reduction. Therefore, if the degeneration of the wall strength is taken into account, the net effect of ILT on AAA wall rupture may vary.

### Change of wall stiffness

Clinical observations show that that most AAA walls become progressively stiffer as the diameter increases. This is because of biomechanical restructuring of the wall. For example, He et al. [37] investigated the composition and mechanical properties of AAAs, and they found that AAA walls were stiffer and volume fractions of collagen and ground substance levels were higher, whereas the volume fractions of elastin and muscle tissue were relatively low. MacSweeney et al. [38] also indicated that the increasing aneurysmal aorta stiffness was associated with the loss of elastin. AAA stiffness is usually correlated with gender and age, as described by Lanne et al. [39]. Enhanced wall stiffness is not necessarily advantageous for preventing AAA rupture, because along with the increase of wall stiffness, the wall yield stress will accordingly decrease. As a case in point, Raghavan et al. [14] stated that Young's modulus in an AAA wall may reach 4.66 MPa, which is about three times that in normal arterial walls; whereas its yield stress is only 50% of the normal artery. Also, Groenink et al. [40] reported that even though stiffness may become large with age, the yield stress of the wall will decrease significantly with respect to age. Thus, although Young's modulus may reduce AAA-wall stress, the yield stress is possibly lower than the mechanical stress in the AAA wall, i.e., AAA rupture still may occur when the wall becomes stiffer. In case wall stiffness decreases with time because of a failure in wall restructuring, the risk of rupture increases significantly. For example, Wilson et al. [23] found that a 10% decrease in stiffness over time was associated with a 28% increase in rupture risk when compared to AAAs without wall stiffness changes. They also indicated that from the time of AAA observation to elective repair, the wall stiffness appears to increase, while the wall stiffness in ruptured AAAs is less than that in elective repaired AAAs. It implies that there is a maximum stiffness before rupture. They declared that the reason of fast expansion before rupture is due to the failure of restructuring; but the actual factors that determine at what point AAA restructuring fails are unknown. Thus, the change of wall stiffness may be a strong player in AAA rupture.

### Saccular index

The saccular index, , i.e., the ratio of maximum AAA diameter to the length of AAA region, is another novel system parameter to express AAA characteristics. Clinical observations indicate that the smaller the saccular index the higher is the possibility of AAA rupture. For example, Ouriel et al. [41] reported that the longer aneurysms may be more dangerous than shorter ones, where the clinical thresholds of saccular indices for elective repair and rupture are 0.6 and 0.7, respectively. Based on clinical data, Hatakeyama et al. [19] studied the influence of saccular index on AAA rupture. Their results demonstrated that the saccular index correlated significantly to AAA rupture. They also fitted an equation to express rupture in terms of saccular index, diameter ratio and diastolic pressure. Li [42] graphed AAA-wall stress vs. saccular index and illustrated that there are two "turning points" at *γ *= 0.65 and *γ *= 0.72. The Von Mises stress increases slowly when the saccular index changes from 0.72 to 0.65. However, once the saccular index is less than 0.65, the Von Mises stress increases quickly. It suggests that the saccular index is another possible predictor of AAA rupture.

## Methods

In order to determine when it becomes necessary to repair AAAs via open surgery or endovascular aneurysm (EVAR) repair, accurate assessment of the risk of AAA rupture is most important. As mentioned, AAA rupture is a complicated multi-factorial event. In an attempt to solve this problem, a new method to predict AAA rupture risk is outlined in terms of a time-dependent, patient-specific severity parameter, 0≤*SP(t)*≤1.0. This dimensionless indicator is composed of eight biomechanical factors (*BF*s) which appeared in the clinical/biomedical literature as most (or very) important (see Table 1). Specifically, the normalized *BF*s in Table 1 represent the maximum diameter ratio, expansion rate, stress ratio, diastolic pressure, asymmetry index, ILT ratio, wall stiffness, and saccular index. The magnitude of the resulting severity parameter is divided into four levels, i.e., low (0.1), middle (0.3) high (0.7), and dangerous (1.0). Each *BF*_*i *_, *i *= 1.......8, has its own weighting coefficient and threshold value based on clinical observations obtained from the open literature as well as recommendations made by endovascular surgeons [23,41,42]. The parameters in Table 1 are described as follows:

**Table 1:** Severity parameters for AAA rupture

***BF*_*i *_**	**Definition**	**Reported ranges of biomechanical factors *(BF_i _)***				**Weighing coefficients w_*i *_**	**References for *BF*_*i *_-ranges**
				
		Low risk	Middle risk	High risk	Danger zone		
*i *= 1: Diameter ratio		1.5~1.9	2.0~2.4	2.5~3.2	≥3.3	0.20	Cappeller [6]
*i *= 2: Expansion rate (per year)		0.01~0.04	0.05~0.09	0.10~0.17	≥0.18	0.25	Limet [2]
*i *= 3: Stress ratio		1.5~2.0	2.1~3.0	3.1~4.3	≥4.4	0.15	Fillinger [4]
*i *= 4: Diastolic pressure ratio		0.83~0.9	0.91~1.0	1.1~1.16	≥1.17	0.12	Cronenwett [20]
*i *= 5: Asymmetry index		1~0.9	0.7~0.8	0.5~0.6	≤0.4	0.07	Vorp [36]
*i *= 6: ILT-AAA ratio		0.1~0.24	0.25~0.44	0.45~0.61	≥0.62	0.07	Cappeller [6]
*i *= 7: Stiffness decrease (per year)		0.01~0.03	0.04~0.06	0.07~0.09	≥0.1	0.07	Wilson [23]
*i *= 8: Saccular index		≥0.71	0.66~0.70	0.61~0.65	≤0.6	0.07	Ouriel [41]

Assigned *BF*_*i *_threshold values		0.1	0.3	0.7	1.0		

(1) *d*_*AA *_is the diameter of the aortic neck.

(2)  is the maximum AAA diameter measured at the previous interval, typically a year. If not available, it can be estimated as follows. Based on clinical data provided by Bernstein et al [43], a correlation for the diameter expansion rate was curve-fitted as:



Thus, the previous diameter is then:



(3a) *σ*_*AAA*,max _, the maximum wall stress is calculated with Eq.(1) given below; clearly, if the patient's actual yield stress would be known, *σ*_*yield *_- *σ*_max _would be the only predictor necessary for AAA rupture.

(3b) The stress in the infrarenal artery is



where *t*_*AA *_is the wall thickness of the infrarenal artery.

(4) *P*_*dia *_is the diastolic pressure (mmHg).

(5) 

*l*_a _is the distance from canter O to the posterior

(6) *Ep *is the pressure strain elastic modulus given as,



where *d*_*max*,*sys *_and *d*_*max*,*dia *_are the maximum AAA diameter under systolic and diastolic condition, respectively;  is the pressure strain elastic modulus in previous measurement. If it is unavailable, 150 kPa was assumed for the first measurement calculation.

**Figure 1:**
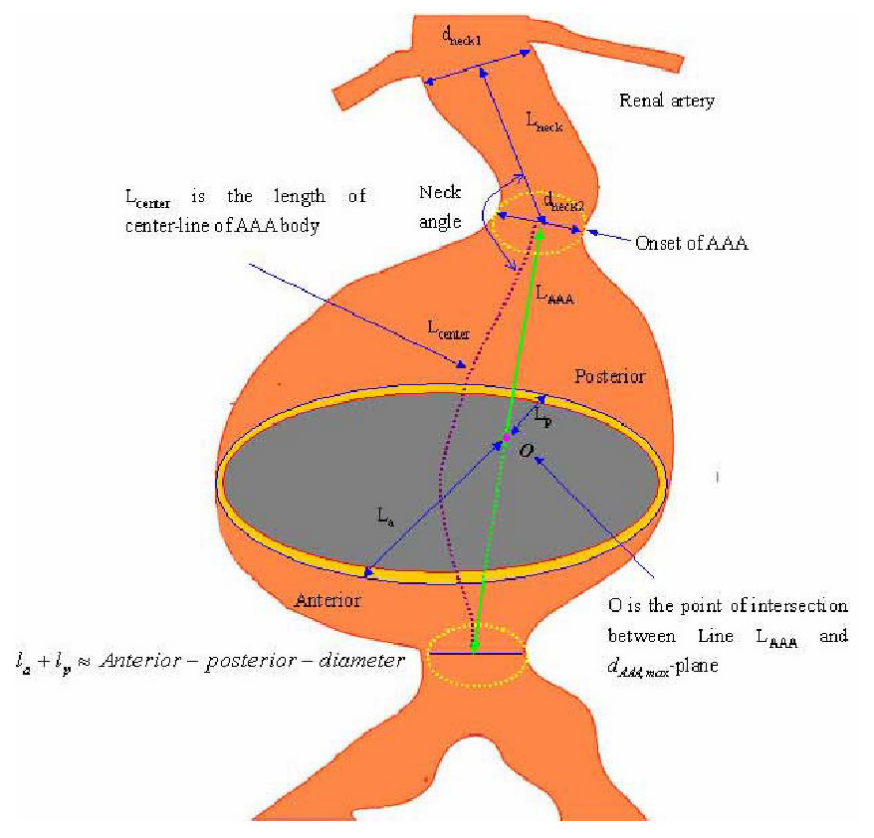
Parameter measurements of abdominal aortic aneurysm for AAA-rupture risk evaluation.

(7) *L*_*AAA *_is the AAA length as shown in Fig. 1.

Once all the biomechanical factors are calculated and threshold values are assigned to each *BF*_*i *_(see Table 1), a severity parameter, SP(t), can be calculated for each patient. Clearly, most elusive, but very important, is the maximum AAA-wall stress. Based on clinical observations reported in the literature and in-house computer simulations, a semi-empirical wall stress correlation has been developed [44]. In essence, we obtained a modified Laplace Equation via multi-parameter curve-fitting:



where the area ratio, the asymmetry index  (See Fig. 1), *P*_*sys *_is the systolic blood pressure (mmHg); *d*_*AAA*,max _is the maximum AAA diameter (cm); and *t *is the wall thickness at the *d*_*AAA*,*max *_location (cm). Specifically, *A*_*AAA *max _and *A*_*ILT*,max _are the transverse areas of the AAA and intraluminal thrombus (ILT) at the *d*_*AAA*,*max *_location, respectively. For Ultrasound and CT scans without area measurement, the transverse area *A*_*AAA*,max _can be approximately calculated as:



where H is the axis normal to *d*_*AAA*,max _(Fig. 1). The lumen area *A*_*lumen*,max _may be calculated similarly; then the ILT area is given as

*A*_*ILT*,max _= *A*_*AAA*,max _- *A*_*lumen *max _    (3)

It can be seen from Eq.(1), the modified Laplace Equation not only represents the nonlinear correlation between wall stress and blood pressure, diameter, and wall thickness, but it also takes into account the effects of an intraluminal thrombus (ILT) and asymmetry. Compared with the simple Laplace Equation, it may provide more accurate estimates for AAA-wall stresses with approximation error less than 10% for AAAs with regular morphology (saccular or fusiform).

As mentioned, the severity parameter (*SP*) is expressed as the summation of each factor multiplied by a corresponding weighting coefficient *w*_*i *_, which reflect the relative importance of the eight *BF*s. Specifically:



After the SP is calculated, the degree of risk and associated recommendation may be obtained from patient-specific SP values. For example, if the overall severity parameter is below 0.2, the risk of rupture is very low; however close observation is required if the severity parameter ranges from 0.2 to 0.45. When the severity parameter is greater than 0.45, elective repair should be seriously considered. If *SP>*0.70, possible rupture may occur and immediate surgical intervention would be necessary. Clearly, if *SP*>0.45 *and *certain symptoms occur, such as back pain, abdominal pain, syncope or vomiting, elective repair should be considered as well.

The procedure for the AAA monitoring program runs as follows:

(1) Obtain basic AAA information of the patient's AAA using Ultrasound or CT scanning.

(2) Calculate each *BF*_*i *_, using Table 1 and related equations.

(3) Obtain the *SP *as the summation of each *w*_*i *_*BF*_*i *_product with Eq.(4) and determine the risk level, i.e., 0< SP(t) <1.0.

(4) Consider other related issues such as symptoms and the patient's special requirement, weigh the risk of operation (i.e., open surgery vs. EVAR) and the computed rupture probability.

## Results and discussion

### Comparisons

A comparison between numerical analyses, the modified Laplace Equation, and the original Laplace Equation for 10 different clinical and numerical AAA models is summarized in Table 2[44].

**Table 2:** Comparison between modified and original Laplace Equations

AAA model	P (mmHg)	*d*_*AAA*,*max *_(cm)	Thickness *t *(cm)	*α*	*β*	Stress (Authors' results) (MPa)	Modified Laplace Equation		Original Laplace Equation	
							
							Stress (MPa)	Error	Stress (MPa)	Error
Fillinger [3,4]	120	6.7	0.19	0	0.4	0.32	0.34	6.3%	0.28	13%
	130	5.5	0.19	0	0.4	0.30	0.28	6.7%	0.25	16.7%
Wang [29]	128	6.1	0.184	0.54	0.33	0.19	0.208	9.5%	0.28	47%
	155	6.4	0.175	0.29	0.9	0.35	0.34	2.9%	0.38	8.6%
Vorp [36]	120	6.0	0.15	0	0.3	0.33	0.34	3%	0.31	6%
Raghavan [45]	115	5.2	0.19	0	0.65	0.23	0.21	8.7%	0.2	13%
	188	5.5	0.19	0	0.9	0.43	0.46	6.9%	0.36	16%
Thubrikar [15]	120	5.86	0.104	0	0.5	0.37	0.39	5.4%	0.45	22%
	120	5.86	0.158	0	0.5	0.3	0.299	0.3%	0.29	3.3%
Li [44]	120	5.0	0.05	0.15	1	0.43	0.41	4.6%	0.80	85.6%

### Monitoring program for AAA-rupture risk

This program is designed to monitor eight risk factor changes for individual AAA-patients, expressed in terms of a dimensionless, normalized, time-dependent severity parameter SP(t). Specifically, based on a patient's measured pressure, AAA geometry and mechanical properties as well as systemic information, the program calculates biomechanical risk factors, evaluates the severity parameter for AAA rupture, and provides recommendations for endovascular surgeons. For example, this clinically tested program can detect AAA-rupture risk in patients with AAA parameters well below *d*_*AAA*,max _= 5 cm and Δ*d*_*AAA*,max _/year = 15%, as commonly used.

**Figure 2:**
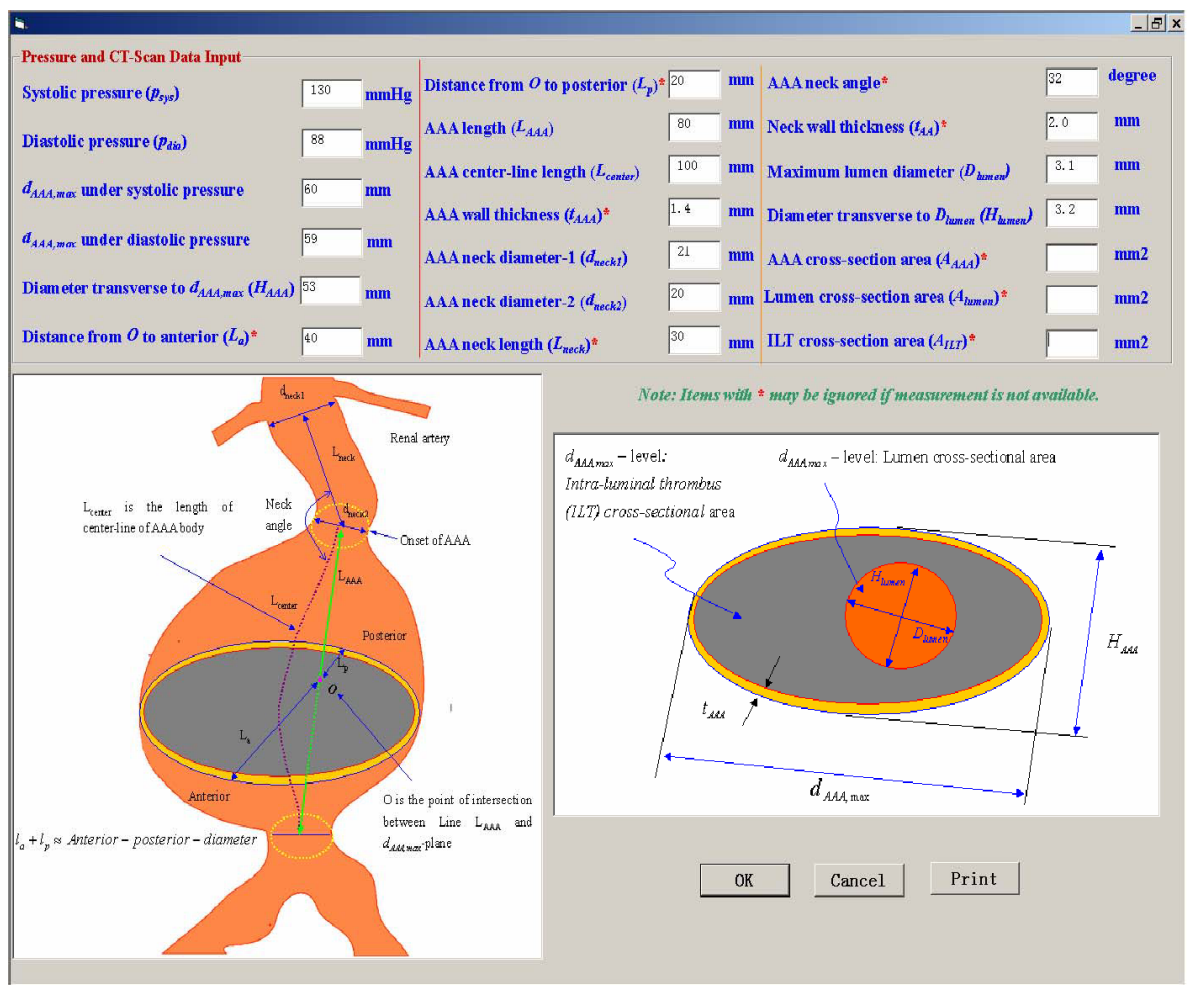
Measured input data requirements.

**Figure 3:**
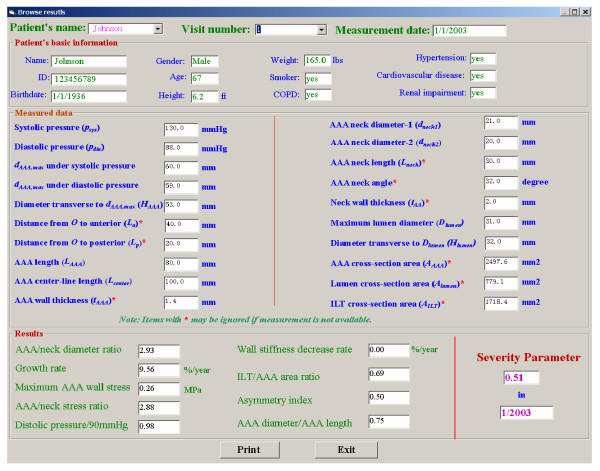
Table of analyzed results.

**Figure 4:**
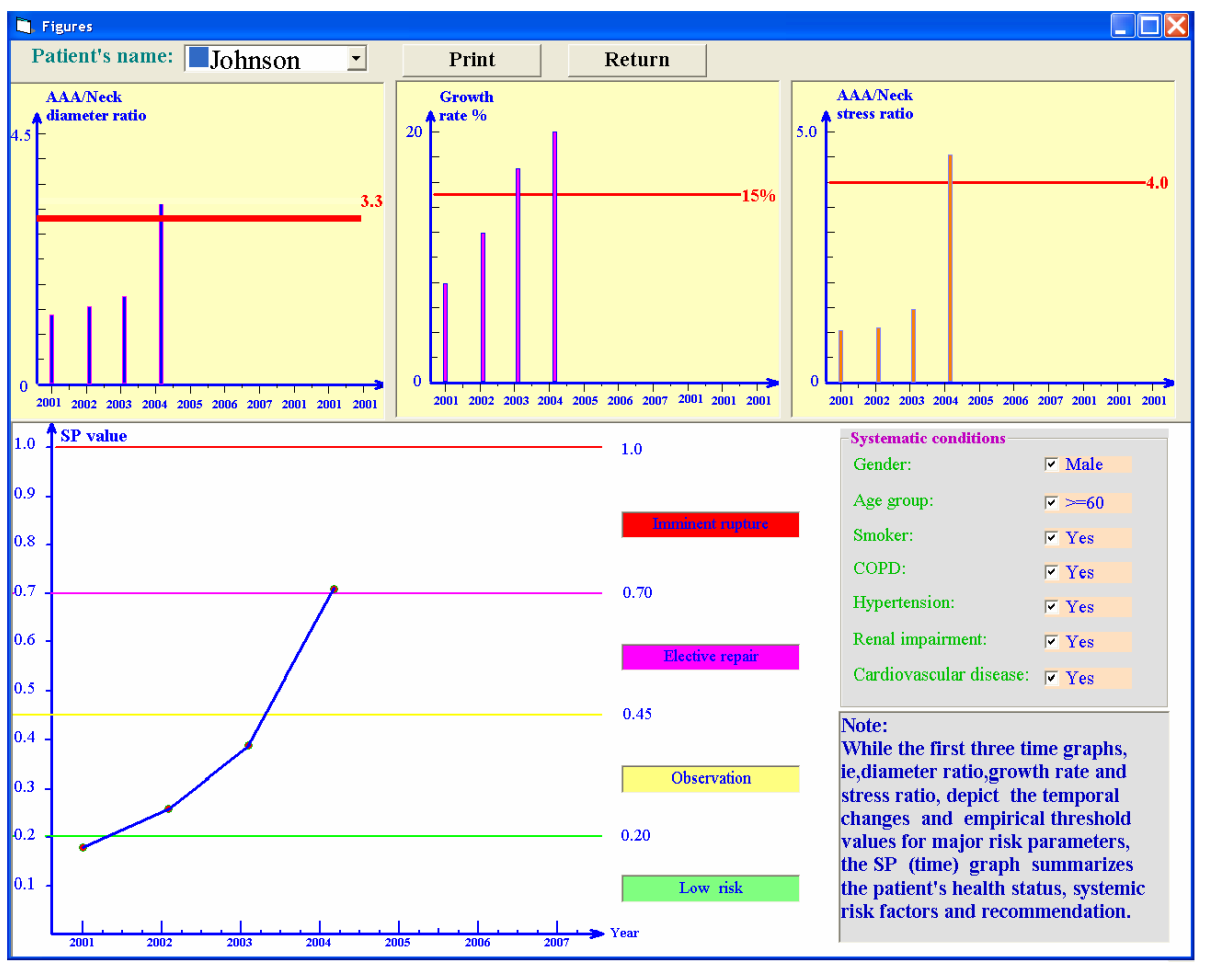
Example of Patient I.

**Figure 5:**
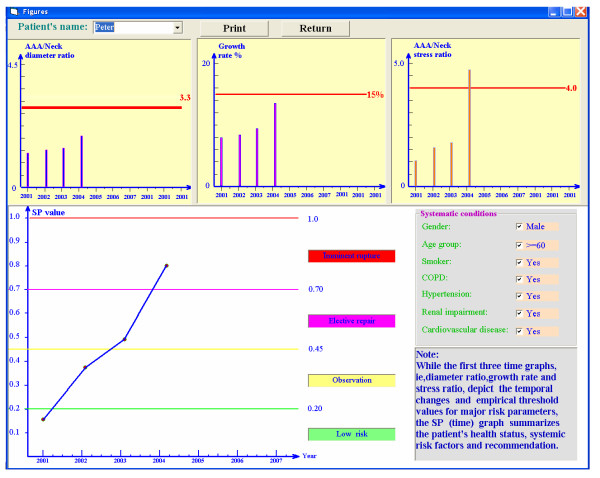
Example of Patient II.

Figures 2, 3 illustrate the clinical data input for the program. The results are demonstrated in Figs. 4, 5. For example, the patient in Fig. 4 experienced in 2004 rapid health deterioration due to accelerated AAA growth (see first two bar graphs), accompanied by a sudden increase in wall stress (see third bar graph). While the critical status of Patient "Johnson" could have been also detected with simple ultrasound measurements of the maximum AAA diameter, the present program provides seven additionally values of important biomechanical factors, making up the severity parameter SP(t). Although falling into the same gender, age, and health group as Patient "Johnson", "Patient Peter" was in mortal danger in 2004 because of the high risk of AAA rupture (see Fig. 5). Interestingly enough, that critical status could not have been detected by just relying on the maximum AAA diameter and the annual AAA-growth rate (see first two bar graphs). The third bar graph reveals that Mr. Peter's AAA-wall stress had exceeded the critical threshold values and rupture was imminent. Clearly, the other biomechanical factors contributed to the SP(t) graph as well.

### Validations

To test the validation of the AAA severity parameter, we selected three clinical AAA cases as documented by Raghavan et al. [45], Wang et al. [29], and Wilson et al. [23], summarized in Table 3.

**Table 3:** Validation of severity parameters for AAA rupture prediction

Reference	Model #1 [45]	Model #2 [29]	Model #3 [23]
Systolic pressure (mmHg)	188	128	150
Diastolic pressure (mmHg)	90	88	85
Maximum AAA diameter (cm)	5.5	6.1	6.36
AAA length (cm)	10.8	8.4	10*
AAA wall thickness (cm)	0.19	0.18	0.2*
Diameter expansion (cm/year)	0.43	0.54	0.61
Diameter of infrarenal artery (cm)	2.0	2.03	2.0
Asymmetry index *β*	0.9	0.33	N/A
ILT-AAA area ratio *α*	N/A	0.54	N/A
Stiffness decrease (% per year)	N/A	N/A	0.21
Severity parameter (SP)	0.5	0.6	0.75
Risk level	Elective repair	Elective repair	Possible rupture
Patient status (clinical)	Waiting for repair	Waiting for repair	Ruptured

* The clinical data were not available and hence typical values were assumed.

The AAA geometries, mechanical properties and hemodynamic factors are very different for the three models. Nevertheless, the SP-value for each case classifies the "patient status" correctly. Some contrasting biomechanical factors are worth mentioning. For example, although Model #1 includes a case of serious hypertension, its severity parameter is 0.5. Model #2 exhibits a large *d*_*AAA*,*max *_, a high expansion rate and a significant asymmetry index, so that its severity parameter value is relatively high 0.6. Thus, both AAAs are located in the region of elective repair, which was verified by the patient's status of "waiting for repair". Although the *d*_*AAA*,*max *_difference is not significant between Model #2 and Model #3, the risk level of Model #3 is much higher because it has a higher systolic pressure (causing high wall stress), a large expansion rate and a serious decrease-rate in wall stiffness, while the ILT in Model #2 reduces the wall stress to some extent. As indicated in Table 3, the AAA Model #3 falls into the category of "possible rupture", which has been clinically confirmed.

In summary, unlike other predictors of AAA rupture, the new methodology is based on eight important biomechanical factors or events potentially leading to AAA rupture. Furthermore, the numerical *BF*_*i *_-values required in this method are relatively easy to measure or to calculate. Clearly, the new methodology is powerful when identifying high-risk AAA patients with ***d*_*AAA*,*max *_*<***5 cm, an occurrence which is possible as confirmed by several researchers [2,4,46].

### Program limitations

There still exist limitations inherent in the present model. For example, the modified Laplace Equation is an approximation for asymmetric AAAs without seriously distorted shapes and large stress concentrations (i.e., caused by large curvature, severe local atheroma or calcification). The material is assumed to be linear, elastic, and isotropic. Actually, an exact wall thickness is difficult to obtain with current measurement approaches due to the thrombus and surrounding tissues. Raghavan et al. [16] indicated that wall thickness may vary regionally between AAAs from as low as 0.23 mm at a rupture site to 4.26 mm at a calcified site. Wall thickness was slightly lower in the posterior and right regions. The error and error propagation of wall thickness measurements will affect the values of maximum stress and accordingly SP value. Furthermore, hypertension and genetics are possible causes of AAA formation; but, their association with AAA rupture is still under investigation. Fillinger et al. [27] reported that smoking is significantly related to rupture, even when adjusting for gender and AAA anatomy. Gender alone is probably a risk parameter because AAA- rupture risk in women is higher than in men. Additionally, as atherosclerotic aneurysms show degenerative changes, coagulative and fibrinolytic functions take part in the process of aneurismal formation. It is well known that local stress exceeding its yield stress is the ultimate cause of AAA rupture. However, the exact stress is difficult to obtain with current measurement techniques, while the accurate local wall strength is unknown as well. Because of these high prevailing uncertainties, only a 15% significance has been assigned to the current SP assessment. If the wall stress/strength can be predicted accurately, it will be considered in an updated SP program. Finally, the weighting coefficients were determined based on consultations with endovascular surgeons and from literature reviews. Such values can be readily updated as additional clinical statistics and verifications are made available.

## Conclusion

The following conclusions can be drawn from this study:

(1) AAA rupture is a complicated and multi-factorial event, dependent upon the maximum diameter, expansion rate, diastolic pressure, wall stress and strength, asymmetry, saccular index, intraluminal thrombus (ILT), and stiffness change, among others. Clearly, one cannot rely on one or two simple factors alone to determine the risk of rupture accurately.

(2) The basic Laplace Equation cannot provide satisfactory estimates for the wall stress in actual AAAs with non-cylindrical geometry. However, a modified Laplace Equation, which encapsulates nonlinear characteristics as well as the effects of ILT and shape asymmetry, may provide convenient and relatively accurate results for wall stress analyses.

(3) The present AAA monitoring program relies on a time-dependent, dimensionless, normalized severity parameter SP(t), which employs eight biomechanical factors and associated weighting coefficients for AAA-rupture risk assessment. It provides a more accurate prediction for individual patients than relying on the maximum AAA diameter and/or AAA growth rate, as conventionally used.

(4) Predicting the rupture of small AAAs is very important but quite difficult. The present SP(t) model, as part of the AAA patient monitoring program, is a new methodology for reliable, patient-specific AAA-rupture risk assessment.

## Authors' contributions

CK conceived the idea of a dimensionless Severity Parameter and developed the necessary biomechanical factors, the modelling equations, and methodology for program testing. CK also wrote the manuscript, while ZL performed the literature review, data collection, programming, and comparisons. Both authors read and approved the final manuscript. CK contributed 75% and ZL contributed 25% to this work. ZL is now working at Endovascular Division, Cordis Corporation (a Johnson & Johnson Company), Miami, FL, USA.
